# Quinacridones as a Building Block for Sustainable Gliding Layers on Ice and Snow

**DOI:** 10.3390/ma17143543

**Published:** 2024-07-17

**Authors:** Peter Bützer, Marcel Roland Bützer, Florence Piffaretti, Patrick Schneider, Simon Lustenberger, Fabian Walther, Dominik Brühwiler

**Affiliations:** 1 Isantin GmbH, 9450 Altstätten, Switzerland; 2 Institute of Chemistry and Biotechnology, Zürich University of Applied Sciences (ZHAW), 8820 Wädenswil, Switzerland

**Keywords:** quinacridone, 2,9-dimethylquinacridone, gliding layer, supramolecular, self-assembly, ice, snow, water, sustainable, QSAR, abrasion, friction

## Abstract

Quinacridone (QA) and 2,9-dimethylquinacridone (DQA) are synthetic substances suitable as a hard, abrasion-resistant, self-organizing gliding layer on ice and snow. For sustainable use, a large number of parameters must be considered to demonstrate that these non-biogenic substances and their by-products and degradation products are harmless to humans and the environment in the quantities released. For this task, available experimental data are used and supplemented for all tautomers by numerous relevant physical, chemical, toxicological and ecotoxicological estimated values based on various Quantitative Structure Activity Relationship (QSAR) methods. On the one hand, the low solubility of QA and DQA leads to stable gliding layers and thus, low abrasion and uptake by plants, animals and humans. On the other hand, the four hydrogen bond forming functional groups per molecule allow nanoparticle decomposition and enzymatic degradation in natural environments. All available data justify a sustainable use of QA and DQA as a gliding layer. The assessment of the toxicological properties is complemented by an investigation of the size and morphology of DQA particles, as well as field tests indicating excellent performance as a gliding layer on snow.

## 1. Introduction

Fluorinated ski waxes, long favored for their exceptional performance in reducing friction on snow, have come under scrutiny and have been increasingly regulated or banned due to their environmental and health hazards [1,2,3]. These waxes contain per- and polyfluoroalkyl substances (PFAS), which are highly persistent in the environment, thus contaminating water sources and impacting wildlife. The ban on fluorinated ski waxes underscores a critical need for developing sustainable alternatives that do not compromise on performance while ensuring environmental safety. The first prerequisite for the successful application of a substance as a sustainable gliding layer for winter sports equipment is a very low coefficient of friction. The second and long-term prerequisite is to provide evidence-based, transparent proof demonstrating the low impact of the substances’ by-products, impurities and degradation products on health and the environment. Expanding upon our previous studies on indigo as a gliding layer [4], we have investigated synthetic quinacridones. In our comprehensive review of existing patents on glide layers (waxes) for skis and snowboards, we observed a diverse range of substance classes utilized to enhance performance. Notably, our search revealed that quinacridones are not listed among the substances currently considered for this application.

Quinacridone was first prepared by Niementowski in 1896 [5], and another yellow quinacridone compound was reported by Ullmann and Maag in 1906 [6]. In 1935, Liebermann published the synthesis of linear trans-quinacridones starting from dimethyl succinyl succinate–based 2,5-dianilinoterephthalic acid [7]. Trans-quinacridones were first sold as pigments by DuPont in 1958 [8] and are considered high-performance pigments because of their exceptional color and weather fastness. The use of bio-succinic acid [9,10] as a greener alternative to traditional petroleum-based succinic acid is established nowadays in the industrial production of quinacridone pigments.

**Figure 1:**
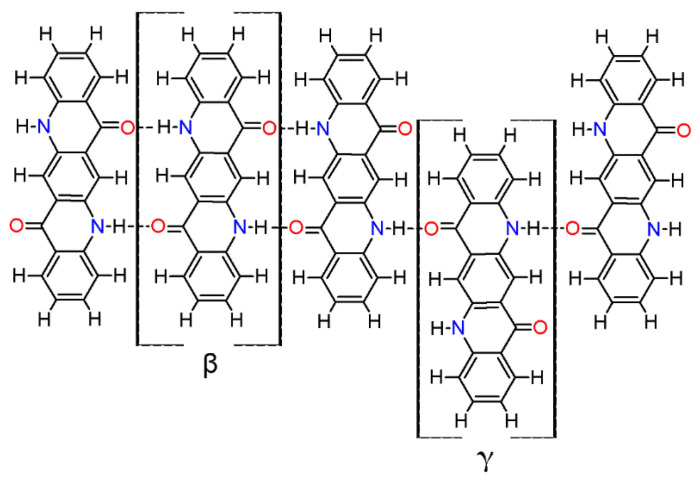
Self-assembly of quinacridone (QA). (**Left**): β-modification with two hydrogen bonds between molecules of the same 2D-chirality. (**Right**): γ-modification with one hydrogen bond between molecules of different 2D-chirality β and γ. The β structures have a higher density than the γ structures [15]. For use as a gliding layer, it has proven useful that the β-modification can be obtained from the more stable γ-modification by tempering up to 200 °C and the use of solvents [16].

Quinacridones form fine crystalline solids based on self-assembly via intermolecular hydrogen bonding (Figure 1) [11,12]. In the isolated molecules, the electrons of the rings are weakly conjugated via the C=O and N-H groups. In the solid state, hydrogen bonds are formed, weakening the C=O and N-H bonds and strengthening the conjugation in the aromatic rings [13]. Quinacridone layers adhere strongly to surfaces. The difference in the maximum desorption temperatures of multilayers of quinacridone (510 K) and pentacene (400 K) underlines the importance of intermolecular hydrogen bonds [14].

In the following, the toxicological properties of quinacridone (QA) and 2,9-dimethylquinacridone (DQA) are assessed, taking into consideration the potential presence of tautomers, impurities and degradation products as well as nanoparticle-associated toxicity. These results are complemented by field tests, demonstrating the suitability of quinacridones as gliding layers on snow.

## 2. Materials and Methods

Quinacridone (QA) and 2,9-dimethylquinacridone (DQA) were purchased from Santa Cruz Biotechnology (Dallas, TX, USA) and Kremer Pigmente (Aichstetten, Germany). Organic solvents were obtained from Sigma-Aldrich. All chemicals were used as received. For the quinacridone dispersions, only pure liquids (>99%) were utilized, which have such a high vapor pressure that few residues (<0.01%) remain when the gliding layer is applied and dried. Scanning electron microscopy (SEM) images were acquired with a Quanta FEG 250 (Thermo Fisher Scientific, Waltham, MA, USA). Electronic absorption spectra were recorded with a Lambda 650 (Perkin Elmer, Waltham, MA, USA). Particle size distributions were measured with a Mastersizer 3000 (Malvern Panalytical, Worcestershire, UK). The field tests on snow consisted of six pairs of calibrated cross-country skis. Each pair was run four times with an optimized test sequence. The glide time on a slightly inclined slope delimited by two light barriers was measured with simultaneous recording of meteorological and snow parameters [17].

## 3. Assessment of the Toxicological Properties of Quinacridones

### 3.1. Comparison of Quinacridones and Biogenic Acridone

The toxicological and ecotoxicological differences between synthetic quinacridones and biogenic acridone are small according to the values in Table 1, especially when the corresponding water solubilities are taken into account. Given the very low water solubility of QA and DQA, these molecules are unlikely to dissolve or be transported easily in aquatic environments. Note that the concentrations of all predicted effects are higher than the water solubility.

### 3.2. Applications of Quinacridones

Quinacridones are organic semiconductors (p-type) [30], used since 1984 for organic photovoltaic cells [31,32]. Furthermore, it has been shown that DQA can provide excellent electrochemical performance as an anode material in lithium batteries [33]. In these types of applications, the purity of the quinacridones is a critical parameter [34]. The current main applications are as pigments in printing, as well as in automotive and industrial coatings.

The very low solubility of QA and DQA represents a significant limitation for chemical–physical–toxicological–ecotoxicological analysis and the assessment of effects on humans and the environment. According to the FDA, DQA may be used as a colorant in food contact polymers at a maximum level of 1.5% by weight of the finished polymer [25]. The authorized use of QA (Pigment Violet 19) and DQA (Pigment Red 122) in cosmetics [35] and for tattoos [36,37] indicates that human toxicity is classified as low. For organic pigments, there is no evidence that other particle properties, such as surface area or morphology, influence the toxicological endpoints [38]. Due to their insolubility in most organic solvents and water, quinacridone pigments are essentially non-bioavailable and therefore are not absorbed or metabolized [39,40].

### 3.3. Quinacridone Particles: Application as a Gliding Layer

Quinacridone nanoparticles can form platelets that cover a surface, where π-π-interactions lead to the formation of multiple layers [41,42]. Such hydrophobic platelets adhere well to many surfaces [43] and form hard, thin layers that have a low coefficient of friction as a gliding layer on ice and snow. A gliding layer of quinacridones is lipophilic because it is wetted by heptane with very small contact angles. In the case of an application as a dispersion on winter sports equipment, the uptake of dispersants and small particles via the lungs is crucial for the assessment of a possible hazard; in the case of organic substances, immune-mediated pulmonary inflammation is of primary importance.

The structure of the gliding surface formed by platelets of quinacridones is determined by their purity, particle size and particle shape, as well as the dispersing solvent and the friction during application. The formation of extended platelets requires that the substances are used in high purity (>97%). By heating and intensive polishing, the quinacridone molecules can be somewhat aligned, and the gliding friction is measurably reduced. The free polar groups at the periphery of the quinacridone platelets do not significantly reduce the hydrophobicity if the supramolecular structures are made up of numerous building blocks. The platelets are classified as granular biopersistent nanomaterials without substance-specific toxicity (GBP nanomaterials) due to their biostability, which is attributed to their water solubility being below 100 mg/L. For the assessment of exposure to GBP quinacridone nanoparticles, an assessment standard of 0.5 mg/m^3^ applies to the alveolar fraction (at an average agglomerate density of 1.5 g/cm^3^ and a mass fraction of 20% nanoscale GBP) [44]. When assessing health hazards from GBP nanomaterials, the focus is on a chronic, inflammatory effect in the lungs after inhalation.

In terms of bonding, hazardous nanoparticles are typically characterized by ionic (e.g., TiO_2_) or covalent bonds (e.g., graphene, carbon nanotubes, microplastics and tire wear). Nanoparticles that are primarily stabilized by hydrogen bonds are typically less robust under physiological conditions. The disruption of the hydrogen bonds ideally leads to the breakdown of the particles before any negative effects due to potential nanoparticle-associated toxicity can occur. This is also likely to be true for the supramolecular assemblies of quinacridones. For QA, the Derived No-Effect Level (DNEL) is established for inhalation (local: 3 mg/m^3^, systemic: 3 mg/m^3^ [45]). The DNEL represents an estimate of the exposure level to a substance below which no adverse health effects are expected to occur over a person’s lifetime. The WHO recommendations for air quality to minimize health risks to the general population are <10 μg/m^3^ annual mean and <25 μg/m^3^ daily mean (for PM2.5, i.e., particulate matter that is 2.5 μm in diameter or smaller) [46]. Considering the low reactivity of quinacridones [47] and their currently known toxicological properties [48], it can be concluded that critical effects are unlikely.

### 3.4. QSAR Data of DQA Tautomers

For a comprehensive assessment of the chronic toxicity and ecotoxicity of quinacridones, a detailed investigation of Quantitative Structure-Activity Relationship (QSAR) data is required in addition to the currently available experimental data. No direct experimental data are available for tautomers of quinacridones, at best, some properties in different solvents. However, with the application of QSAR methods, it is possible to estimate properties for the individual tautomers. Since the various QSAR methods differ in their descriptors, a range of results is obtained, as shown in Table 2.

Even for pure synthetic chemical substances, a detailed assessment of the degradation products is necessary to exclude the presence of highly active substances that could cause problems. Therefore, the following by-products and degradation products were considered: 2,5-diaminobenzene-1,4-dicarboxylic acid; 2,5-dianilinoterephthalic acid; 2,5-di-p-toluidinoterephthalic acid; 2-amino-5-methylbenzoic acid; 5-methylanthranilic acid; 3-hydroxyacridin-9(10H)-one; 6-hydroxy-2-methylacridin-9(10H)-one; aniline; anthranilic acid; 2-aminobenzoic acid; benzene-1,4-diol; hydroquinone; benzoic acid; p-toluidine and terephthalic acid.

Hydrogen bond forming, self-assembling substances such as indigo, QA and DQA are structurally so exclusive that no analogous structures can be found with the Analog Identification Methodology (AIM) software in the Toxic Substance Control Act Test Submission (TSCATS) database [49]. This is confirmed by the structural differences in 225 records of similar compounds, based on the Jaccard similarity coefficient > 0.8 [50], and in the sections (5 related records) in PubChem [25] for the individual substances. It is also confirmed for substructures such as 10H-acridin-9-one (CAS No. 578-95-0), 1H-quinolin-4-one (CAS No. 529-37-3), 1,6-dihydropyrido[2,3-g]quinoline-4,9-dione, 2-methyl-10H-acridin-9-one (CAS No. 23864-43-9) and 6-methyl-1H-quinolin-4-one (CAS No. 23432-40-8). No QSAR model can account for intra- and intermolecular hydrogen bonding. As a consequence, the water solubility of quinacridones cannot be accurately estimated. Therefore, estimates of collective properties with QSAR models provide data with large uncertainties.

The different tautomers of quinacridones are present depending on the environment, but the experimental data of the individual tautomers are not directly measurable. To ensure that individual tautomers do not exhibit critical properties, their data were estimated with QSAR models and compared with all other possible tautomers and evaluated in absolute terms. Table 2 shows a selection of values for DQA tautomers. One consequence of the tautomerism of quinacridones is the strong hydrogen bonds resulting from the formation of resonance-assisted hydrogen bonds (RAHB), which are also found in DNA/RNA [51].

**Table 2:** QSAR values for 2,9-dimethylquinacridone (DQA) tautomers. The variations in the characteristic properties of the tautomers indicate possible different behaviors in different environments (complete list: [52]).

	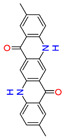	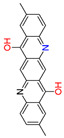	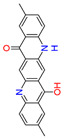	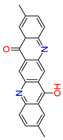	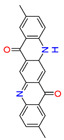
Number of aromatic atoms ^d^	12	22	20	14	12
sp^3^sp^2^ hybridization ratio ^d^	0.136	0.0909	0.0909	0.136	0.136
Density [g/cm^3^] ^a^	1.31 ± 0.06	1.41 ± 0.06	1.36 ± 0.06	1.38 ± 0.1	1.38 ± 0.1
Molar volume [cm^3^] ^a^	260.3 ± 3.0	241.7 ± 3.0	251.0 ± 3.0	246.3 ± 7.0	246.3 ± 7.0
Polarizability [10^−24^ cm^3^] ^a^	38.82 ± 0.5	42.50 ± 0.5	40.66 ± 0.5	39.10 ± 0.5	39.10 ± 0.5
Surface tension [dyne/cm] ^a^	54.3 ± 3.0	77.0 ± 3.0	65.7 ± 3.0	53.3 ± 7.0	53.3 ± 7.0
Total polar surface area ^e^	58.2	66.2	62.2	62.0	58.3
pK_a_^d^	6.4	5.1	8.3	5.4	6.4
log(Kow) ^b^	4.1	4.8	3.6	4.7	4.1
log(BCF) ^d^	1.33	1.76	1.53	1.69	1.33
Water solubility [mg/L] ^b^	2.2	0.046	0.44	0.056	2.2
NOAEL [mg/kg bw] ^c^	20.6	26.8	11.0	28.3	20.6
Acute toxicity (LD_50_) [mg/kg bw] ^d^	2244	2747	3170	623	2244
Persistence (sediment) [days] ^c^	229.1	70.8	49.0	229.1	229.1
Persistence (soil) [days] ^c^	33.9	22.9	4.9	33.9	33.9
Persistence (water) [days] ^c^	26.3	4.2	3.9	22.4	26.3
Sewage treatment plant total removal ^b^	34.83%	69.53%	16.34%	65.05%	34.83%
Sludge (EC_50_) [mg/L] ^c^	31.13	23.92	32.01	35.09	31.13
Estrogen Receptor-mediated effect ^c^	NON active (all tautomers)				
Estrogen Receptor activity, binding ^d,f^	0 (all tautomers)				
Estrogen Receptor activity, agonist ^d,f^	0 (all tautomers)				
Estrogen Receptor activity, antagonist ^d,f^	0 (all tautomers)				
Androgen Receptor-mediated effect ^c^	NON active (all tautomers)				
Androgen Receptor activity, binding ^d,f^	1 (all tautomers)				
Androgen Receptor activity, agonist ^d,f^	0 (all tautomers)				
Androgen Receptor activity, antagonist ^d,f^	1 (all tautomers)				
Thyroid Receptor alpha effect ^c^	Inactive (all tautomers)				
Thyroid Receptor beta effect ^c^	Inactive (all tautomers)				
Endocrine disruptor activity screening ^c^	Inactive (all tautomers)				

^a^ ACD/ChemSketch [53]; ^b^ EPI [54]; ^c^ VEGA [29]; ^d^ OPERA [55]; ^e^ OSIRIS [26]; ^f^ ToxCast [50].

### 3.5. Experimental Data

Laser-induced degradation of different pigments identified one or more of the carcinogens hexachlorobenzene, 3,3’-dichlorobenzidine, aniline and benzene in most cases, but no degradation products were detected for DQA [37,56]. The oxidative degradation of quinacridones leads to the toxicologically and ecotoxicologically uncritical benzoic acid [57]. Biodegradation is expected to be slow [25]. QA does not enter living cells [58]. Some cases of contact dermatitis or hypersensitivity reactions to quinacridones as tattoo inks have been reported, but patch testing did not induce an allergic reaction [59,60]. This is supported by the experimental data [61,62]. In the repeated dose toxicity study according to OECD TG 422, reproductive and developmental toxicity, no effects of DQA on clinical signs were observed, so the No Observed Adverse Effect Level (NOAEL) and the No Observed Effect Level (NOEL) were 1000 mg/kg/day [63]. Important experimental data for DQA are as follows [24]:

- Density: 1.452 g/cm^3^ (20 °C);
- Water solubility: 5.6 µg/L (24 °C);
- Log(Kow): 2.2;
- Short-term toxicity to fish: LC_50_ (96 h) > 100 mg/L nominal concentration;
- Long-term toxicity to freshwater fish NOEC: 10 mg/L;
- Bioconcentration Factor (BCF) [64]: 5;
- Daphnia magna chronic NOEC [64]: 1.5 mg/L.

The relevant environmental toxicity and fate limitations on persistent, bioaccumulative and toxic chemicals are as follows: Chemicals with a half-life > 60 days, BCF/BAF > 1000, and toxicity in aquatic environments (i.e., LC/EC_50_ < 10 mg/L or NOEC/LOEC < 1 mg/L) are considered persistent, bioaccumulative and aquatically toxic [65]. The mentioned quinacridones are considered non-bioaccumulative because they have a BCF < 1000, a log(Kow) < 5 [50] and a maximum environmental persistence of 2 months [66]. For the repeated dose toxicity (RDT) test [67], 14% of the QSAR-estimated NOAEL can be achieved for QA and 8% for DQA due to their low solubility.

Although initially characterized as a cellular receptor regulating toxicological responses to xenobiotic compounds, the aryl hydrocarbon receptor (AHR) plays essential roles in human development, normal organ function and metabolic homeostasis [68]. AHR deficiency or dysregulation underlies the pathophysiology of several disease states, including circadian rhythm disruption, myocardial hypertrophy and intestinal barrier dysfunction. The structural configuration of quinacridone as a polynuclear heterocyclic aromatic compound and its derivatives as a polynuclear aromatic hydrocarbon would suggest binding to the AHR. Based on the chemical structure, a comparison with indigo and indirubin seems reasonable [69]. The important factor with this binding is that the effects are not negative as with structurally comparable agonist analogs [70,71,72,73]. It must also be ensured that the possibility of an effect as an endocrine disruptor [74] can be excluded as far as possible. The quinacridones cannot be assigned to any class listed in reference [75].

Supramolecular quinacridone clusters are degradable, unlike graphene, whose covalent bonds make the nanoparticles hardly degradable and therefore toxic [76,77]. Even carbon black is possibly carcinogenic to humans [78]. Therefore, it is necessary to show that critical chemical [79], toxicological and ecotoxicological properties differ significantly from those of pentacene [80] and carbon black. The comparison of pentacene with quinacridone shows an important difference, namely the four additional functional groups. Functional groups such as ketones and amines are critical for enzymatic degradation as they enhance substrate recognition, binding and reactivity, support specific enzymatic mechanisms, increase biodegradability and facilitate integration into metabolic pathways. All of these factors contribute to reduced ecotoxicity as compounds are more efficiently and completely degraded in the environment.

## 4. Characterization of DQA Particles and Performance as a Gliding Layer

### 4.1. Purity and Electronic Absorption Spectra

The purity of quinacridones is of crucial importance for their sustainable use, as adverse toxic effects could be attributed to harmful impurities [38]. The following reaction partners or impurities of the synthesis were analyzed: 2,5-dihydroxyterephthalic acid; aniline; dimethyl succinate; dimethyl succinyl succinate (DMSS); dimethyl-2,5-dianilino-3,6-dihydroterephthalate; methanol; p-toluidine and succinic acid. Chemical analysis is difficult because reaction partners of the synthesis might be trapped as by-products or impurities in the poorly soluble particles. Toxic and ecotoxic aniline and p-toluidine were identified as the most critical by-products of the synthesis of QA and DQA, respectively. The quantification by HPLC resulted in a value above the detection limit but below the determination limit.

**Figure 2:**
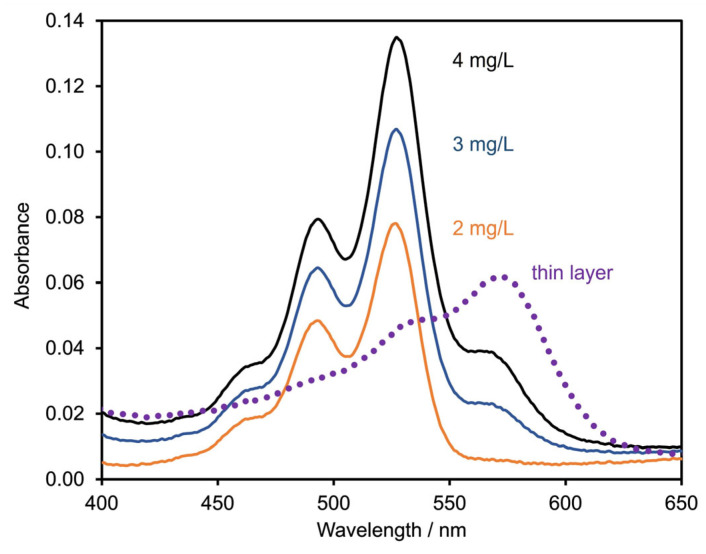
Electronic absorption spectra of 2,9-dimethylquinacridone (DQA) in dimethylformamide (DMF) at three different concentrations (solid lines). The shoulder at about 565 nm indicates the formation of molecular aggregates. The spectrum of a thin layer of DQA on glass is shown as a dotted line.

The acquisition of electronic absorption spectra of DQA is similarly challenging due to the low solubility. Dilute solutions (concentration below approx. 6 μmol/L) in DMF feature the expected fine-structured absorption band with a maximum at 527 nm (Figure 2). The shoulder at 565 nm, which intensifies with increasing concentration, is an indication of the pronounced tendency towards the formation of molecular aggregates [81]. In order to determine the electronic absorption properties of the gliding layer, a thin layer of DQA was deposited onto a glass substrate and measured in transmission. Supramolecular structures held together by hydrogen bonds are most likely responsible for the observed red shift. The absorption maximum of the thin layer corresponds well with the absorption band of the molecular aggregates in the solution.

### 4.2. Particle Size and Morphology

**Figure 3:**
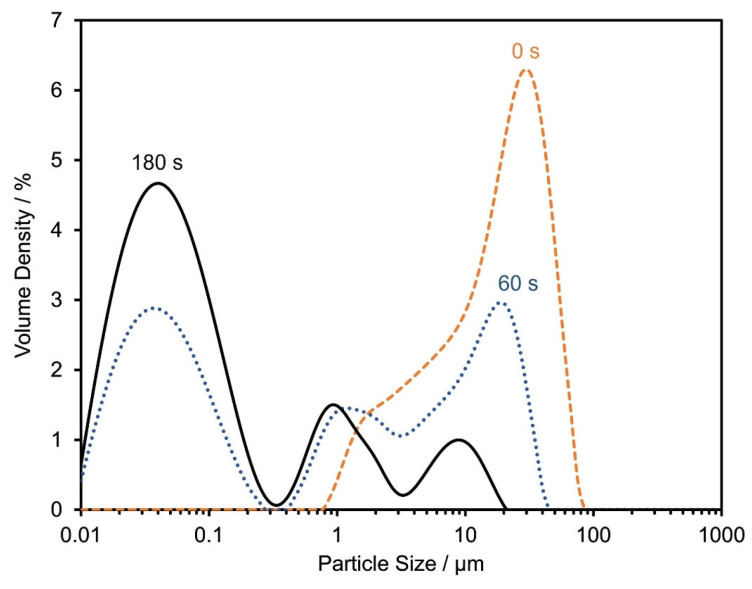
Particle size distribution of DQA in ethanol, after 0 s (dashed), 60 s (dotted) and 180 s (solid line) of ultrasonic treatment.

**Figure 4:**
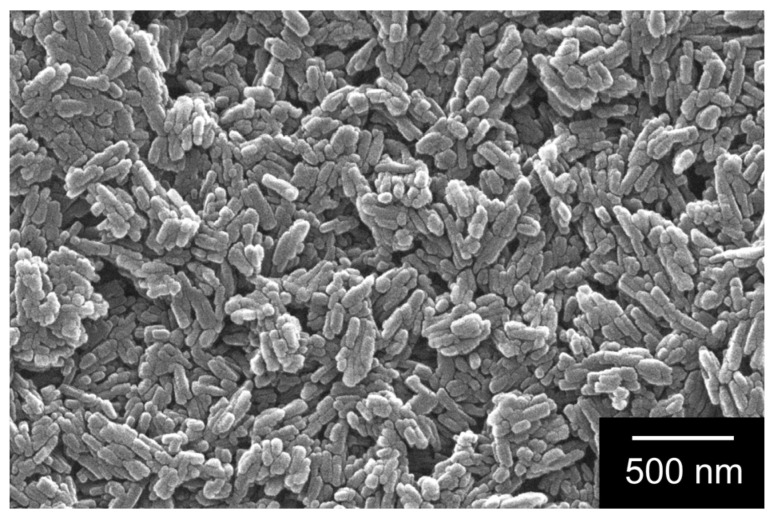
Scanning electron microscopy image of DQA particles.

The particle size distribution has a decisive influence on the technical performance of the application as a gliding layer. Ultrasonic treatment of a dispersion of DQA in ethanol shows that the initial micrometer-sized agglomerates are readily broken up into primary nanoparticles (Figure 3). The electron microscope image shown in Figure 4 suggests that the dry powder of DQA indeed consists of agglomerated nanoparticles.

**Figure 5:**
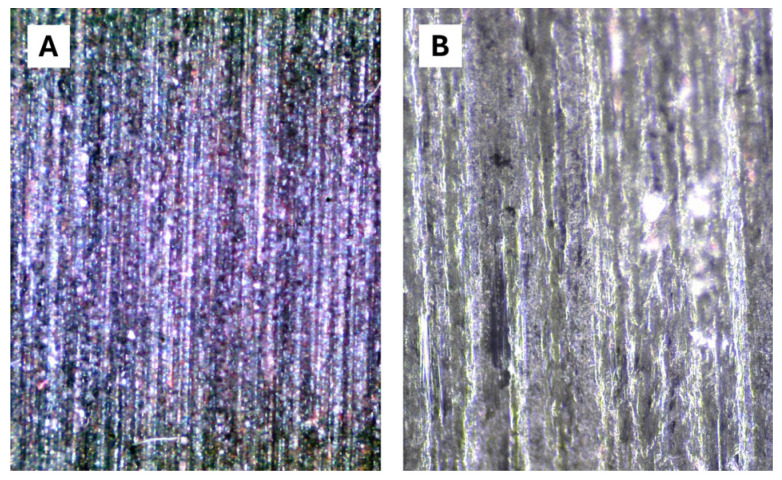
Micrographs (magnification 50× (**A**) and 650× (**B**)) of a ski base (UHMWPE with graphite-additive, cross-country ski, Atomic GenS with factory grind) coated with a layer of DQA. The differences in color are due to strong reflections.

The microscopic images (Figure 5) show that the ski surface (factory grind) can be coated with a layer of DQA without much loss of surface structure. The prerequisite for this, however, is that the DQA dispersion is applied several times and is rubbed in with high pressure so that the nanoparticles can assemble into a supramolecular gliding layer by self-organization.

Modern ski bases are typically made from ultra-high molecular weight polyethylene (UHMWPE) with surface filament ends forming a fiber carpet. This fiber carpet must be integrated into the smooth gliding layer to achieve low coefficients of friction. Measurements have shown that the hardness (DIN 51579-65) of linear paraffin (C24–C25) increases from 22 [82] to 32–37 after the addition of 33% (*w*/*w*) DQA. In comparison, a hard ski wax (blue) for cold temperatures has a hardness of 27–30. The surface hardness achieved with DQA is, therefore, particularly suitable for cold, hard snow.

### 4.3. Performance

Quinacridones form ice-repellent surfaces similar to polydimethylsiloxanes (e.g., [83]) when applied as a top coat. Water droplets brought to freeze on a polystyrene surface or on a DQA layer on polystyrene bind less strongly to DQA and do not pull DQA with them when the water droplet is pushed away. In this context, indigo should be mentioned as a similar self-organizing stable substance but with slightly different lubricating properties [4]. Quinacridones are intended to be used as a gliding layer at temperatures below −1 °C, where liquid water can no longer form on the surface of snow or ice [84,85].

**Figure 6:**
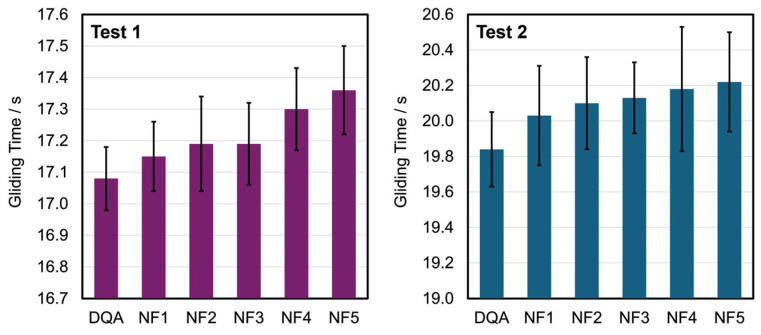
Running times over a measuring section with two photoelectric barriers for different ski gliding layers. The mean values are the result of four measurements, each with calibrated cross-country skis (old snow, 0.5–0.8 mm, no dirt; see Table 3 for the conditions). NF—non-fluorinated wax. DQA—2,9-dimethylquinacridone.

The field tests presented in Table 3 and Figure 6 indicate that DQA is particularly suitable for snow temperatures below −6 °C. Estimates for large events, such as the Engadin ski marathon, show that the abrasion of DQA (<0.1 mg/km per person), distributed in the air as dust and absorbed through respiration, remains below the lowest known legal limits. In comparison, the abrasion of car tires as toxic fine dust of the tread ranges from 55 to 212 mg/km per car [86]. During test weeks involving various participants, different ski brands and changing snow conditions, we observed no white spots, which would indicate complete abrasion, on the ski base after at least 50 km.

## 5. Conclusions

It is a particular challenge to use synthetic quinacridones in a sustainable product with convincingly transparent and sufficiently comprehensive data. The experimental data, which have been very sparse to date, need to be supplemented with physical, chemical, toxicological and ecotoxicological values from QSAR assessments to enable a broad, evidence-based evaluation. This is particularly important for quinacridones because, as tautomers, these molecules can adapt to their local environment and therefore exhibit variable properties that are difficult to access experimentally.

Quinacridone and 2,9-dimethylquinacridone can be readily formed into a self-assembled supramolecular gliding layer and exhibit low coefficients of friction and low abrasion on ice and snow. The low residue levels and low bioconcentration in the environment, coupled with the poor solubility, result in low doses in biochemical cycles (plants, animals, humans) and, together with low acute and chronic toxicity, minimal exposure. The quinacridones form nanoparticles whose toxic effects can be classified as non-hazardous over all exposure routes due to their molecular properties and supramolecular structures. The molecules released from the nanoparticles also show no critical effects on humans or the environment, according to the experimental and QSAR data.

## Figures and Tables

**Table 1:** Comparison of selected characteristic properties of the structurally similar biogenic acridone, quinacridone (QA) and 2,9-dimethylquinacridone (DQA). NOEC—No Observed Effect Concentration; NOAEL—No Observed Adverse Effect Level; DM—Daphnia Magna; Druglikeness—A positive value states that the molecule contains predominantly fragments frequently present in commercial drugs.

	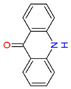	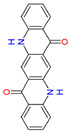	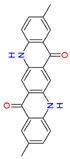
Name	acridone [18]	quinacridone	2,9-dimethylquinacridone
Abbreviation	–	QA	DQA
CAS No.	578-95-0	1047-16-1	980-26-7
Origin	Biogenic [19,20,21]	synthetic	synthetic
Water solubility [mg/L] (24 °C)	0.0047 [22]	0.0103 [23]	0.0056 [24]
log(Kow) ^gm7^	2.6	3.1	4.0
Bioconcentration factor (BCF) ^gm7^	20.5	14.8	26.7
log(Koc) ^gm3^	3.3	5.0	5.1
Green algae EC_50_ (96 h) [mg/L] ^a^	112	293	55.8
Algae chronic (NOEC) [mg/L] ^b^	0.057	0.027	0.025
DM acute EC_50_ [mg/L] ^b,gm2^	0.35	0.55	0.56
DM LC_50_ (48 h) [mg/L] ^b,gm2^	0.97	1.33	1.12
DM chronic (NOEC) [mg/L] ^b^	0.42	1.27	1.10
Fish acute LC_50_ [mg/L] ^b^	10–100	1–10	1–10
Fish chronic (NOEC) [mg/L] ^b^	0.071	0.039	0.028
Sludge EC_50_ [mg/L] ^b^	18.0	26.3	28.9
Earthworm LC_50_ (14 d) [mg/L] ^a^	366	630	529
Bee toxicity [µg/bee] ^b^	>100	>100	>100
LD_50_ (rat, oral) [mg/kg] ^b^; (experimental rat [mg/kg]) [25]	2402	3352 (>20)	2633 (>23)
Endocrine disruptor activity ^b^	inactive	inactive	inactive
Total body elimination half-life [h] ^b^	5.2	8.4	10.8
NOAEL [mg/kg bw]	4.9	6.3	10.9
Druglikeness [26,27]	0.78	0.79	−1.03
Drug Score [26]	0.39	0.14	0.08

^a^ ECOSAR [28]; ^b^ VEGA [29]; ^gmx^ geometric mean calculated from x different QSAR models.

**Table 3:** Conditions of the field tests. The results are shown in Figure 6.

Conditions	Test 1	Test 2
Location	Hoch-Ybrig, Switzerland	St. Moritz, Switzerland
Date	8 January 2021	20 January 2021
Snow temperature [°C]	−8.5	−7
Air temperature [°C]	−8	−2
Air humidity [%]	83	67

## Data Availability

The original contributions presented in the study are included in the article, further inquiries can be directed to the corresponding authors. QSAR data of 2,9-dimethylquinacridone tautomers are available at https://zenodo.org/doi/10.5281/zenodo.8032055 (accessed on 13 June 2023).
